# Comparison of contrast-enhanced ultrasound imaging (CEUS) and super-resolution ultrasound (SRU) for the quantification of ischaemia flow redistribution: a theoretical study

**DOI:** 10.1088/1361-6560/ad9231

**Published:** 2024-11-22

**Authors:** Lachlan J M B Arthur, Vasiliki Voulgaridou, Mairead B Butler, Georgios Papageorgiou, Weiping Lu, Steven R McDougall, Vassilis Sboros

**Affiliations:** 1 School of Engineering & Physical Sciences, Heriot-Watt University, Edinburgh EH14 4AS, United Kingdom; 2 Translational Healthcare Technologies Team, Centre for Inflammation Research, University of Edinburgh, Edinburgh EH16 4TJ, United Kingdom; 3 School of Energy, Geoscience, Infrastructure and Society, Heriot-Watt University, Edinburgh EH14 4AS, United Kingdom

**Keywords:** contrast agent, capillary, pathology modelling, vessel imaging

## Abstract

The study of microcirculation can reveal important information related to pathology. Focusing on alterations that are represented by an obstruction of blood flow in microcirculatory regions may provide an insight into vascular biomarkers. The current in silico study assesses the capability of contrast enhanced ultrasound (CEUS) and super-resolution ultrasound imaging (SRU) flow-quantification to study occlusive actions in a microvascular bed, particularly the ability to characterise known and model induced flow behaviours. The aim is to investigate theoretical limits with the use of CEUS and SRU in order to propose realistic biomarker targets relevant for clinical diagnosis. Results from CEUS flow parameters display limitations congruent with prior investigations. Conventional resolution limits lead to signals dominated by large vessels, making discrimination of microvasculature specific signals difficult. Additionally, some occlusions lead to weakened parametric correlation against flow rate in the remainder of the network. Loss of correlation is dependent on the degree to which flow is redistributed, with comparatively minor redistribution correlating in accordance with ground truth measurements for change in mean transit time, $\textrm{dMTT}$ (CEUS, *R* = 0.85; GT, *R* = 0.82) and change in peak intensity, $dI\textrm{p}$ (CEUS, *R* = 0.87; GT, *R* = 0.96). Major redistributions, however, result in a loss of correlation, demonstrating that the effectiveness of time-intensity curve parameters is influenced by the site of occlusion. Conversely, results from SRU processing provides accurate depiction of the anatomy and dynamics present in the vascular bed, that extends to individual microvessels. Correspondence between model vessel structure displayed in SRU maps with the ground truth was $ > 91\%$ for cases of minor and major flow redistributions. In conclusion, SRU appears to be a highly promising technology in the quantification of subtle flow phenomena due ischaemia induced vascular flow redistribution.

## 1. Introduction

Microcirculation is the transport method responsible for the majority of vital nutrients and oxygen exchange throughout the body. It consists of vessels under 150 *µ*m in diameter that are sensitive to alterations in the local environment, providing vital information about changes in surroundings through vascular remodelling and evolution. The structure and dynamic behaviour of the microcirculation is, thus, linked to the presence of disease. Indeed, various pathologies may be associated with disruption or damage to microcirculatory function including inflammation, cancer, and ischaemia. However, the detection of such vascular effects remains challenging using current imaging technology. Ultrasound imaging is a safe, accessible, and efficient way of reading real-time blood flow information. Doppler ultrasonography , is effective for assessing flow in larger vessels (Hoskins *et al* 2010, Oglat *et al* 2018) but is limited in detecting microvascular flow, as the signal from the blood, primarily from red blood cells, is drowned out by surrounding tissue signal and low values of blood flow are not possible to record (Hoskins 2010). Another modality, contrast-enhanced ultrasound (CEUS) makes use of intravenous contrast agents in the form of microbubbles (MBs) that are composed of an inert gas surrounded by a thin lipid shell. MBs scatter incident ultrasound pulses more effectively than the surrounding medium and can serve as intravascular markers, which can be used to detect changes in the microcirculation (Sboros and Tang 2010). There is an abundance of research from the past 30 years on tissue perfusion quantification that exploits the spatio-temporal evolution of MBs in the vascular bed (Cosgrove *et al* 2001, Sboros and Tang 2010, Strouthos *et al* 2010, Quaia 2011). It relies on image intensity as captured in the ultrasound scanner display macroscopically—with near-millimetre resolution for a typical abdominal ultrasound application—and does not directly enable the differentiation of macro- and micro-vasculature. Several limitations have been identified related to ultrasound equipment, patient variability and contrast stability and preparation (Tang *et al* 2011, Averkiou *et al* 2020). This suggests that the use of CEUS based perfusion quantification is semi-quantitative at best as is the case of other imaging modalities such as MRI and CT.

The advent of super-resolution ultrasound imaging (SRU) promises to resolve such problems by providing direct measurement of the microvascular bed. A number of methods have been developed (Couture *et al* 2011, Siepmann *et al* 2011, O’Reilly and Hynynen 2013, Viessman *et al* 2013, Diamantis *et al* 2019, 2019a, 2019b) including the first *in vivo* pre-clinical studies to show microvessels at sub-wavelength resolution (Christensen-Jeffries *et al* 2015, Errico *et al* 2015, Ghosh *et al* 2017, Lin *et al* 2017, Opacic *et al* 2018). It would be beneficial to evaluate the performance of CEUS and SRU with an *in silico* model that is artefact-free in order to allow a theoretical evaluation of these modalities as macroscopic perfusion quantification methods. Additionally, the deployed vascular model is generated artificially to ensure that all features of the network are well-quantified to perform as an accurate comparator to the subsequent CEUS and SRU interpretations. Further, avoiding network complexity also minimises systematic uncertainty for either technology perfusion quantification, and thus is an efficient way to address their theoretical comparison in isolation. The *in silico* investigation here explores an ischaemia scenario—typically the result of a flow obstruction in a microcirculatory region—and stands as a simplified model of thrombosis, atherosclerosis or an increase in interstitial pressures typically caused by cysts or inflammation. It is known that such a situation affects the neighbourhood of vessels and perfusion to the site of obstruction and, depending on the severity, can also impact a large vascular region and hence the function of an organ. Although obstructions of this size are often asymptomatic or result in significantly milder symptoms than occlusion in larger vessels, they nevertheless represent a potential for gradual development into serious chronic illness (Martini 2006, Seeley *et al* 2008). Therefore, the detection of minor obstructions is directly linked to early diagnosis of several serious conditions. CEUS and SRU have diagnostic functionality, which is compared in the subsequent investigation. Whilst the technical deployment of these modalities is distinct, both aim to address the assessment of perfusion, here linked to ischaemia. Additional value presents in the comparison between the macroscopic perfusion information generated by CEUS, to the cumulative microscopic, single vessel scale information generated by SRU for the same size region.

## 2. Materials & methods

A critical part of the study is the control and measurement of the ground truth (GT) properties of the vasculature (e.g. vessel structure, flow behaviour) in order to validate the imaging quantification. The particular aspect of ischaemia that is investigated here is the vascular remodelling and the models that describe this are coupled with *in silico* phantoms that simulate the imaging. The *in silico* experiments consist of the following stages: (a) vascular network design, (b) simulation of MB/particle transport, (c) ultrasound imaging simulation, and (d) image processing of ultrasound images, either for conventional CEUS or SRU analysis.

### 2.1. Network flow modelling

The *in silico* vascular network designed for this study uses the simulation package *numPTI* (Boujelben *et al* 2016) which includes a variety of perfusion-related features—vessel remodelling, phase separation, metabolic signalling—and has been used successfully in the past to model capillary beds associated with tumour-induced angiogenesis, wound healing, and retinal development (McDougall *et al* 2006, 2012, Macklin *et al* 2009, Machado *et al* 2011, Watson *et al* 2012, Wu *et al* 2013, 2014, Boujelben *et al* 2016). The foundation of the vascular system is represented by an adjustable cylinder with a radius in line with that of microvessels. A connected group of these components constitute the vasculature used in this study. The volumetric flow rate for an individual vessel is based on the generalised Poiseuille’s law:

\begin{align*} Q = \frac{\pi R^{4}\Delta P}{8\mu_\mathrm{app}\left(R,H_{D}\right)L}\end{align*}

where Δ*P* is the pressure drop across the vessel, $\mu_\mathrm{app}$ is the dynamic viscosity of blood, *L* is the length of the vessel, *Q* is the volumetric flow rate, and *R* its radius. Mass conservation considerations at each junction in the network produces a set of linear pressure equations that—together with the application of appropriate inlet and outlet pressure boundary conditions—allows for the solution of the pressure field throughout the domain. Once the pressure field has been determined, equation (1) can be revisited to calculate individual capillary flows. A full description of the flow model can be found in McDougall *et al* (2006). The simulator requires a total inlet perfusion rate as input and the inlet and outlet pressure conditions adapt so that the global pressure drop, and perfusion rate are consistent.

### 2.2. Host tissue vascular design

In order to produce a branching network comprising large arterial inlets and venous outlets connected via daughter vessels of varying radius, the following process was adopted:

(A) A regular 2D network of microvessels was constructed. With capillary radii drawn randomly from a uniform distribution $[1, 120]\mu\mathrm{m}$, the largest 20% of vessels are then enlarged by 25%, giving the vessel range in table 1—this introduces a degree of heterogeneity into the domain prior to adaptive flow. The physical dimensions chosen for the domain are 40 mm × 40 mm.
(B) A degree of distortion was applied to the regular network through the displacement of vessel junctions to create a non-uniform flow gradient. No distortion would result in a uniform flow gradient. Post-adaptation, this would cause an unrealistic, straight preferential flow path. Distorting forces flow from bottom right to top left, ensuring axial and lateral movement of particles throughout the network. Setting distortion too high would result in vessel overlap and thus a sporadic, chaotic flow gradient. As such the distortion value of 30% is chosen, as a result of an iterative testing process, to best represent physiological relevance. That is, high enough to form a central preferential flow path from bottom right to top left, whilst not over distorting such that the pressure gradient becomes chaotic. Vessel segments were randomly removed to reduce the network connectivity (so that, on average, 3 vessels meet at a point of anastomoses). A single high pressure arterial inlet and low pressure venous outlet were introduced and connected to their respective network edges using simple branching structures.
(C) Vessel adaptation was performed by flowing the network using a dynamic process that takes into account intravascular pressures, wall shear stresses, and metabolic/conducted stimuli (Watson *et al* 2012).

**Table 1.** Imaging parameters used in FieldII simulation.

Network and flow parameters	
Minimum vessel radius	$1\,\mu\mathrm{m}$
Maximum vessel radius	$150\,\mu\mathrm{m}$
Mean vessel radius	$135\,\mu\mathrm{m}$
Degree of distortion	30%
Flow rate	$5 \times 10^{-9}$ m^3^ s^−1^
Blood viscosity	1.2 kg m^−1^ s^−1^

**Figure 1.**
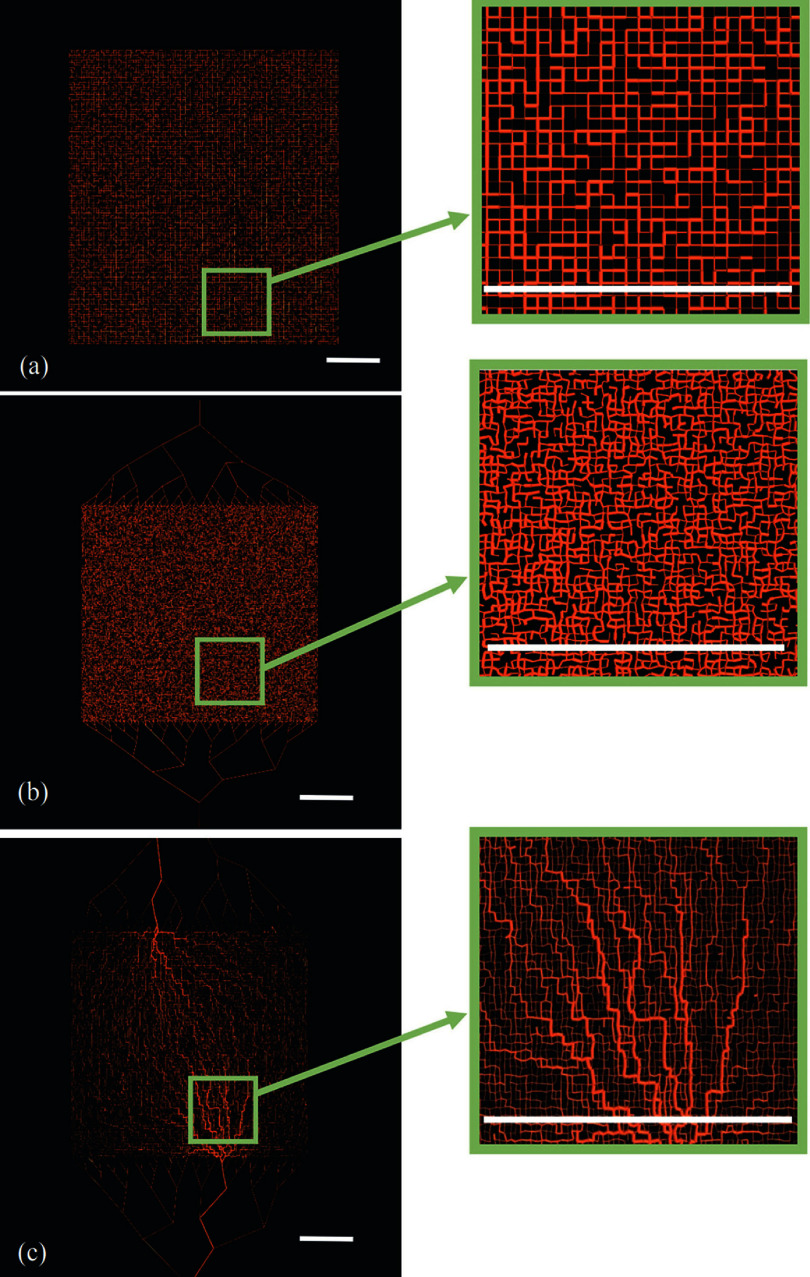
Evolution of network design: (a) uniform, regular lattice, (b) network including inlet and outlet, with applied distortion. (c) Final network, which includes radial adaptation. Vessels are shown in red. Scale bar 10 mm.

Figure 1 shows the various stages of network design described by (A)–(C). The final network is the result of vascular adaptation, and it is this network that will be used as the tissue model for the subsequent studies. Table 1 details the network and flow parameters used for the flow simulations—viscosity and flow were set to reflect typical values found in the microcirculation (Hoskins 2017).

### 2.3. Particle flow simulation, bolus injection, introduction of obstructions

Post-adaptation, the flow rates of vessels are calculated, and dimensionless particles are injected at the inlet of the network and are continuously tracked to simulate CEUS MB transport. Still frames are captured at 20 Hz, which is typical for CEUS, and are used as the phantom input data for the US simulation. The simulation requires a realistic MB injection profile as input. The MB kinetics corresponding to an organ distal from the heart was chosen to facilitate conventional CEUS TIC analyses and so the injection rate of particles was chosen to match those previously conducted on ovine ovaries (Sboros *et al* 2011). In this organ the duration of the bolus passage is approximately 50 s (5%–95% of contrast intensity) and the best fit was provided by the lognormal model (Strouthos *et al* 2010). This was converted to a bolus particle injection rate that peaks at 700 particles per second. For the super-resolution MB detection *in silico* experiment, the MB injections were administered as constant infusions. The global flow rate was the same as with the bolus injection at $5\times10^{-9}$ m^3^ s^−1^ and the injection rate was 5 particles per second: a concentration that ensured adequate sparsity throughout the network that enabled the effective detection of most particles. Model values range from 2–4.5 particles/cm^2^ per frame from wash-in demonstrates sparsity beyond that of the typical bolus concentrations used in Kanoulas *et al* (2019) of 37–106 particles/cm^2^ in an ovary study. The total duration of the MB flow study was 180 s.

**Figure 2.**
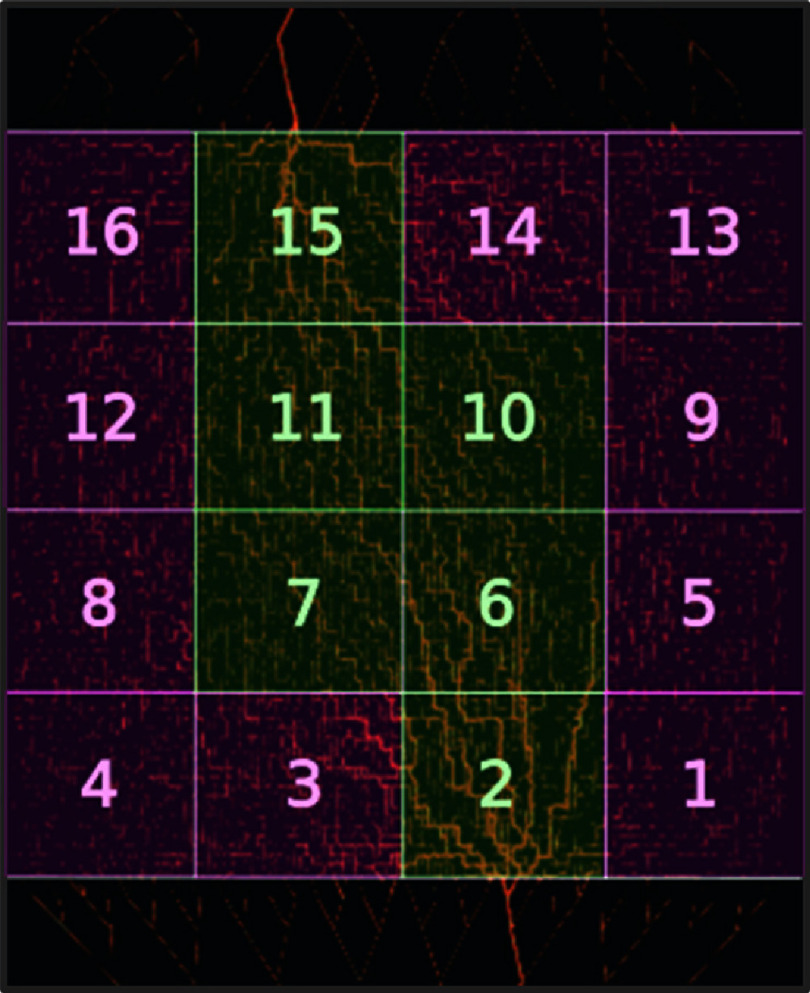
Division of tissue domain into 16 × 1 cm^2^ regions of interest (ROIs). ROIs can be roughly grouped into main-flow regions (denoted in green) and peripheral-flow regions (denoted in magenta).

Flow obstructions were modelled by dividing the vascular bed into 16 equal square sections of dimensions 10 mm × 10 mm, as illustrated in figure 2. The occlusion size was selected to be adequate to measure vascular changes macroscopically and as captured by the resolution of CEUS. CEUS is known to capture such changes at the square cm scale (Tang *et al* 2011), for the specific imaging resolution of a standard radiological application here. In addition, and given the size of the network, the section size allows high sensitivity measurement within the scale of perturbations to vascular flow behaviour both proximal and distal to the occlusion region. In a series of 16 flow studies, a different section was removed from the total network. The absence of each ROI creates a unique flow pattern, and thus a flow redistribution occurs in the remaining network.

### 2.4. CEUS image generation

The perfusion model simulates particles as dimensionless points, that flow through the vessel network. The spatial positions of each particle are output over time can be used as inputs to simulate CEUS images using the simulation package FieldII (Jensen and Svendsen 1992, Jensen 1997). The dimensionless particle flow is upgraded to MB flow by assigning a unique scatter amplitude value to every particle. In the absence of reasonable CEUS simulators, the image generation adopts an adapted B-mode signal processing, where the absence of tissue represents the CEUS tissue cancellation and the highly variable microbubble scatter, due to a varying acoustic field, varying MB physical properties and size, can be accommodated. This is indeed an ideal simulation environment that also reduces CEUS signal processing related uncertainties and further enables the fundamental comparative study of the two modalities. The imaging specifications of the conventional B-mode imaging applied in this study are detailed in table 2. The vascular network is considered to be at 27–131 mm depth, typical for common radiological applications. The focal point is located at 90 mm depth and dynamic receive focusing is used. The dynamic range is set at 40 dB and pixel size is $25\,\mu\mathrm{m}$.

**Table 2.** Imaging parameters used in FieldII simulation.

Transducer information	
Transducer type	Linear
Element pitch	208$\,\mu\mathrm{m}$
Kerf	35$\,\mu\mathrm{m}$
Element height	6 mm
Centre frequency *f*_0_	7 MHz
Sampling frequency $f_\mathrm{s}$	20 MHz
Bandwidth	60%
Wavelength	220$\,\mu\mathrm{m}$
Excitation pulse	2-cycle
Receive apodisation	Conventional
No. of transmit elements/emission	1
Number of receiving elements	198
Number of emissions	198

**Figure 3.**
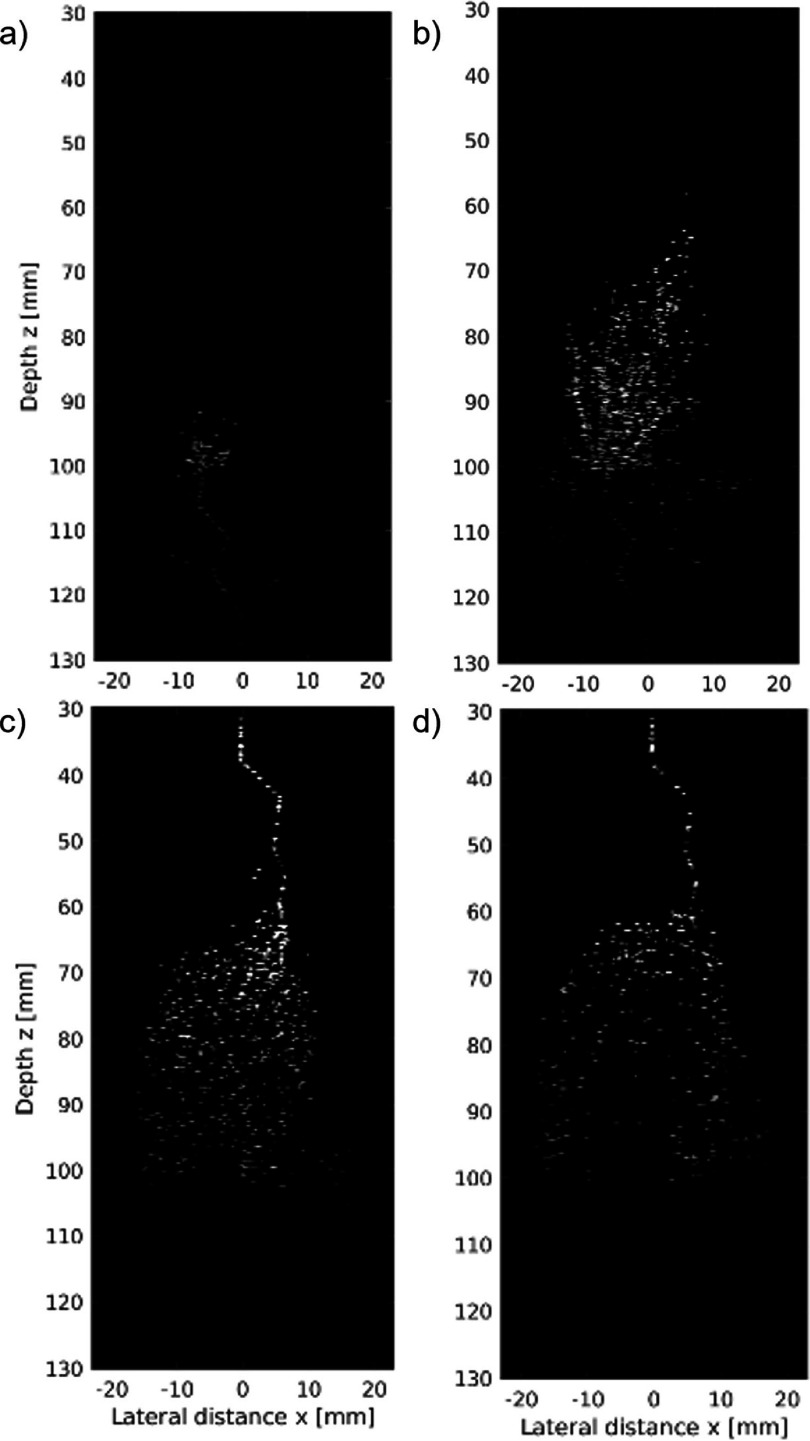
Still frames of MB bolus flow through network figure 1(c) at (a) $t = 5s$, (b) $t = 10s$, (c) $t = 15s$, and (d) $t = 25s$. The MBs are faster and in high concentrations along the main flow path of the network and travel at slower speeds and disperse elsewhere.

A sample of the generated images is shown in figure 3, which represents MB transport through the network shown in figure 1(c). It is already possible to see that MBs flow at higher concentrations along the main flow paths of the organ model as compared to the remainder of the network.

### 2.5. Conventional CEUS image processing

The CEUS time intensity-curves ($\mathrm{TIC}$) resulting from the processing of the video loops are the most common quantitative assessment of network vascular dynamics. This is achieved by using the average intensity of a specific region of interest (ROI) over time, the extent of which can vary in size from a few pixels to the entire image of the network.

Measurable TIC parameters include the peak intensity ($I\mathrm{p}$), time to *Ip* ($t_{\mathrm{p}}$), mean transit time ($\mathrm{MTT}$), wash in time, wash in rate, wash out time, wash out rate, area under curve ($\mathrm{AUC}$), and the regression coefficient to the lognormal function (*R*). Parametric maps were also used to provide blood perfusion related information (Angelelli *et al* 2011, Butler *et al* 2019). The creation of regions of no-flow—or ischaemia—are reflected in the resulting CEUS image datasets. The CEUS data is collected over three different length scales: (i) a single pixel (i.e. parametric mapping), (ii) larger ROI that are similar in size to the ischaemic region (i.e. 1 cm^2^) and (iii) the residual networks left behind after the removal of each ischaemic region. In (ii), for each of the ischaemic regions there are 15 remaining ROIs, each of which lends itself to TIC analysis. The case for which there is no ischaemic region is considered the base/‘healthy’ network case. Figure 2 shows the ROI arrangement for case (ii) - the repetition of the flow problem generates 256 TICs (15 ROIs for each removed ROI plus the single base case comprising all 16 ROIs). In order to assess the CEUS performance, the TIC derived parameters were compared with equivalent parameters directly derived from the model, that constitutes the GT for the experiments. Here, the determination of $\mathrm{TIC}$ parameters utilised log normal functions fit to the particle versus time curves or time particle curves ($\mathrm{TPC}$). The various parameters are then plotted against structural or dynamic properties of the same ROIs—such as average radius and average flow—thereby revealing correlations between parameters ($\mathrm{TIC}$ or $\mathrm{TPC}$ derived) and structural/dynamic properties.

### 2.6. Super-resolution analysis

In addition to the CEUS $\mathrm{TIC}$ analysis, a sub-set of the networks were studied using a MB infusion and particle localisation and tracking methodologies (Kanoulas *et al* 2019). The first was characterised by ischaemia along the main flow path region (removal of ROI6, hereafter referred to as ‘main-flow occlusion’), whilst the second exhibited ischaemia in a region away from the main flow path (removal of ROI8, hereafter referred to as ‘peripheral-flow occlusion’). The flow results were studied for two key areas, one directly downstream of the main-flow occlusion (ROI10) and one directly downstream of the peripheral-flow occlusion (ROI12). The super-resolution maps generated for the study include: number of detections per pixel, track densities, and average velocities. The SRU map results are compared to GT maps, which are generated directly via particle data from numPTI (Boujelben *et al* 2016). Quantitative comparison of SRU velocity maps to GT particle locations and velocity maps enabled a quantitative assessment of SRU performance in recovery of model structure and flow behaviour. SRU quantification enables the direct correlation to the model at individual vessel scales. This is not the case for CEUS, which possesses poorer resolution. As such, the investigation assesses the value of each of the methods with a widely established macroscopic quantification for CEUS metrics and a microscopic one for SRU metrics. The comparison between them is fundamentally performed on the basis of their quantitative value as projected to the specific ischaemia model and its perfusion implication.

## 3. Results

### 3.1. CEUS parametric maps

**Figure 4.**
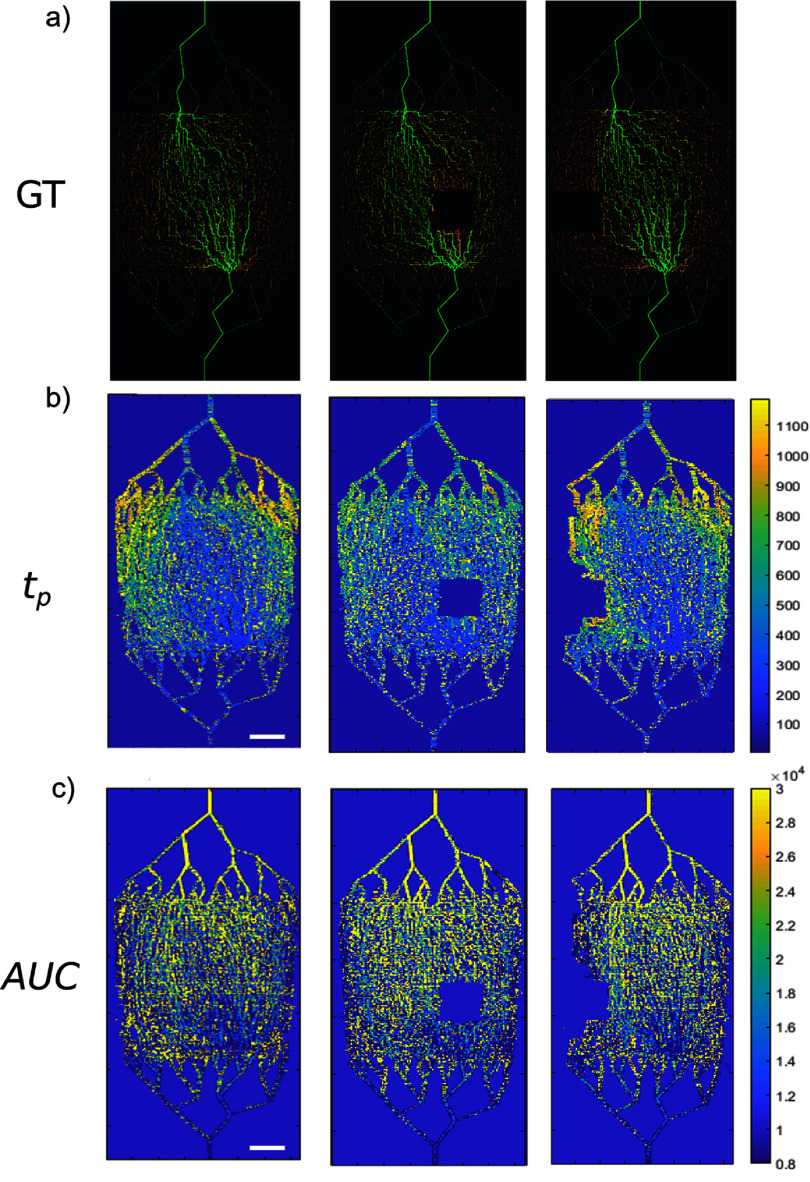
(a) Maps of ground truth flow distribution showing a perfusion of tracer (green) flowing through the network vessels (red) demonstrating the preferential flow path. (b) Parametric maps of time-to-peak ($t_\mathrm{p}$) and (c) area-undercurve ($\mathrm{AUC}$) for the same networks. Every pixel value is the measure $t_\mathrm{p}$ or $\mathrm{AUC}$ from the $\mathrm{TIC}$ of the pixel. The ‘main flow’ path is also visible and mirrors the visible behaviour observed in the GT maps. Scale bar represents 10 mm.

Figure 4 shows how parametric maps of $t_\mathrm{p}$ and AUC (figures 4(b) and (c)) compare with the GT (figure 4(a)). The three columns of imaging are for the base network, for a main-flow occlusion, and a peripheral-flow occlusion respectively. In the $t_\mathrm{p}$ map (figure 4(b)) the lowest values (low $t_\mathrm{p}$ corresponds to high blood velocity) are along the middle where the main-flow path is located (left column images). Little difference is observed in the $t_\mathrm{p}$ map of peripheral-flow occlusion (right column images) where the main-flow path remains clearly visible, with the addition of a boundary effect of negligible flow around the occluded region, confirmed by the GT map (figure 4(a)). The $\textrm{AUC}$ maps (left and right) depict the higher volumetric flow rate only close to the main-flow path, showing less clearly the flow pattern shown in the $t_\mathrm{p}$ maps. On the other hand, the main-flow occlusion maps (figure 4 middle column) depict the significant redistribution of flow, as a general decrease of $t_\mathrm{p}$ throughout the network, and as an increase of $\textrm{AUC}$ only at the top of the network. These characteristics of the parametric maps do not match the detail shown in the GT map (figure 4(a)). One limitation of the method is that the fit to the lognormal model leads to erroneous parameter estimation in 2% of the pixels, often due to a lack of adequate data that does not fit the lognormal function. On the other hand, 91% of the pixels provide regression coefficients $ > 0.99$. A small proportion of these are not a good fit to the lognormal owing to an abundance of data at the wash out. Both these effects may lead to under- or over-estimation of parameter values in the map and should be possible to avoid. However, the fact remains that such processing and image generation has limited resolution and results in an averaging effect that often conceals local dynamic features of the flow network.

### 3.2. Local region of interest analysis

**Figure 5.**
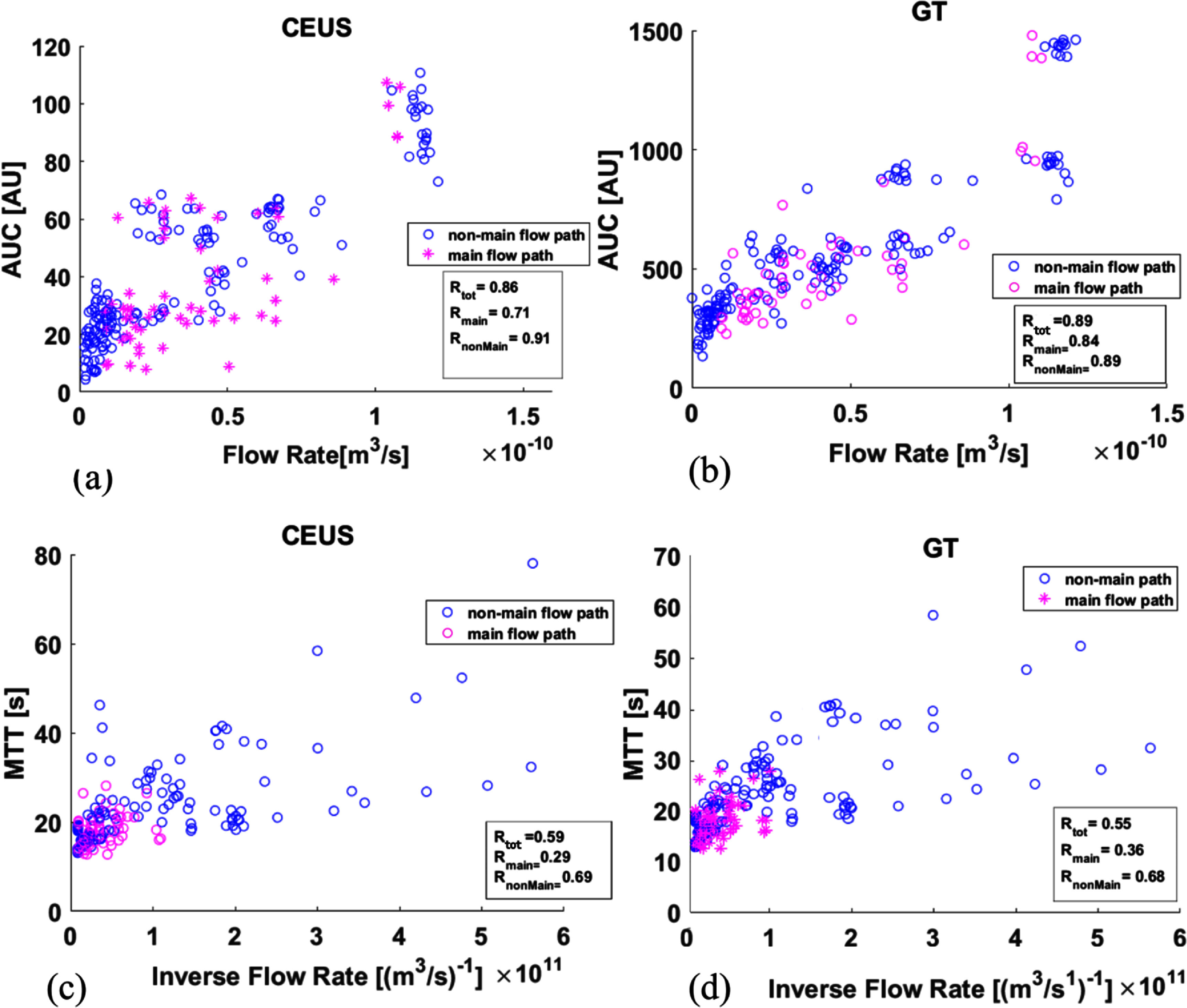
Scatter plots of $\textrm{TIC}$ parameters measured for all the ROIs that have flow vs equivalent GT measurements. (a) $\textrm{AUC}$ of a ROI measured from $\textrm{TIC}$ vs GT flow of the same ROI, (b) $\textrm{AUC}$ of a ROI measured from $\textrm{TPC}$ vs GT flow of the same ROI, (c) $\textrm{MTT}$ of a ROI measured from $\textrm{TIC}$ vs GT inverse flow of the same ROI, (d) $\textrm{MTT}$ of a ROI measured from $\textrm{TPC}$ vs GT inverse flow of the same ROI. The points that correspond to the main-flow path are marked in magenta while the peripheral-flow path points are in blue.

The $\mathrm{TIC}$ analysis in 1 cm^2^ ROIs provided excellent fits to the lognormal function. Figure 5 plots $\mathrm{AUC}$ figures 5(a), (b) and $\mathrm{MTT}$ figures 5(c), (d) for all ROIs, where (a) and (c) represent $\mathrm{TIC}$ data and b and d represent time particle curves ($\mathrm{TPC}$) all against GT measurements. The scatter plots include measurements from the 15 ROIs from all 16 flow networks that result from varying the occluded region position. Also, included are the 16 ROIs of the base network. In figures 5(a) and (b) $\mathrm{AUC}$ data displays a positive correlation with flow rate and in agreement with theory. The points that correspond to the main-flow path are marked in magenta while the peripheral-flow path points are in blue. Occlusions in the main-flow path trigger a flow remodelling that also provides reduced correlation (*R* = 0.71) with volumetric flow rate as compared to peripheral-flow occlusion generated points (*R* = 0.91). This suggests that the redistribution of flow due to main-flow occlusion impacts systematically on regional $\textrm{AUC}$ measurements. Comparing $\textrm{TIC}$ with $\textrm{TPC}$ data shows that the imaging process introduces uncertainty, but most importantly it is the $\textrm{AUC}$ measurement itself that is inherently not adequate in quantifying volumetric flow rate. On the other hand, the $\textrm{MTT}$ scatter plots do not correlate well with the inverse of the flow rate for networks that have main-flow occlusion (magenta markers), conversely there is a significant correlation between $\textrm{MTT}$ and inverse flow rate in network with peripheral-flow occlusions (blue markers) figure 5(c)). These distributions are very similar to the $\textrm{TPC}$ derived data (figure 5(d)). Direct observation of data shows it is due to high flow rates (lower values of inverse flow rate in the *x*-axis) that $\textrm{MTT}$ experiences large variance, suggesting that $\textrm{MTT}$ and transit related physical quantities are inherently limited in providing quantitative measures of average velocity or flow, particularly when these quantities are high or highly variable within a region, which is the case in significant flow redistribution. As found in Butler *et al* (2019), $\textrm{MTT}$ of larger vessels dominate the signal in cases of low transit time and high flow due to the intensity dependence of $\textrm{MTT}$. This obscures signal from surrounding microcirculation and the lack of correspondence in ischaemic regions laid on the main-flow path.

The results show that flow remodelling is sensitive to the location of occlusion. This essentially means that an occlusion in a region of high flow rate would have a greater impact on flow-remodelling compared to an occlusion in a region of lower flow rate. Also shown is that $\textrm{MTT}$ and transit time quantities are not generally accurate measures for capturing such flow changes, while intensity related quantities provide significant uncertainty in the measurement of blood volume or volumetric flow rate. In addition, it is known that these measures are relative and contain information relevant to the dynamics of connected upstream and downstream vasculature. Indeed, the theory that provides the link of $\textrm{MTT}$ and $\textrm{AUC}$ to blood volume and flow rate uses a number of assumptions including conservation of mass and single input/output (Strouthos *et al* 2010), which typically are not held (including the experiment here). All the above are inherent to the macroscopic or cumulative nature of transit and intensity derived measurements as confirmed by the GT $\textrm{TPC}$ data.

### 3.3. Entire vascular bed analysis

**Figure 6.**
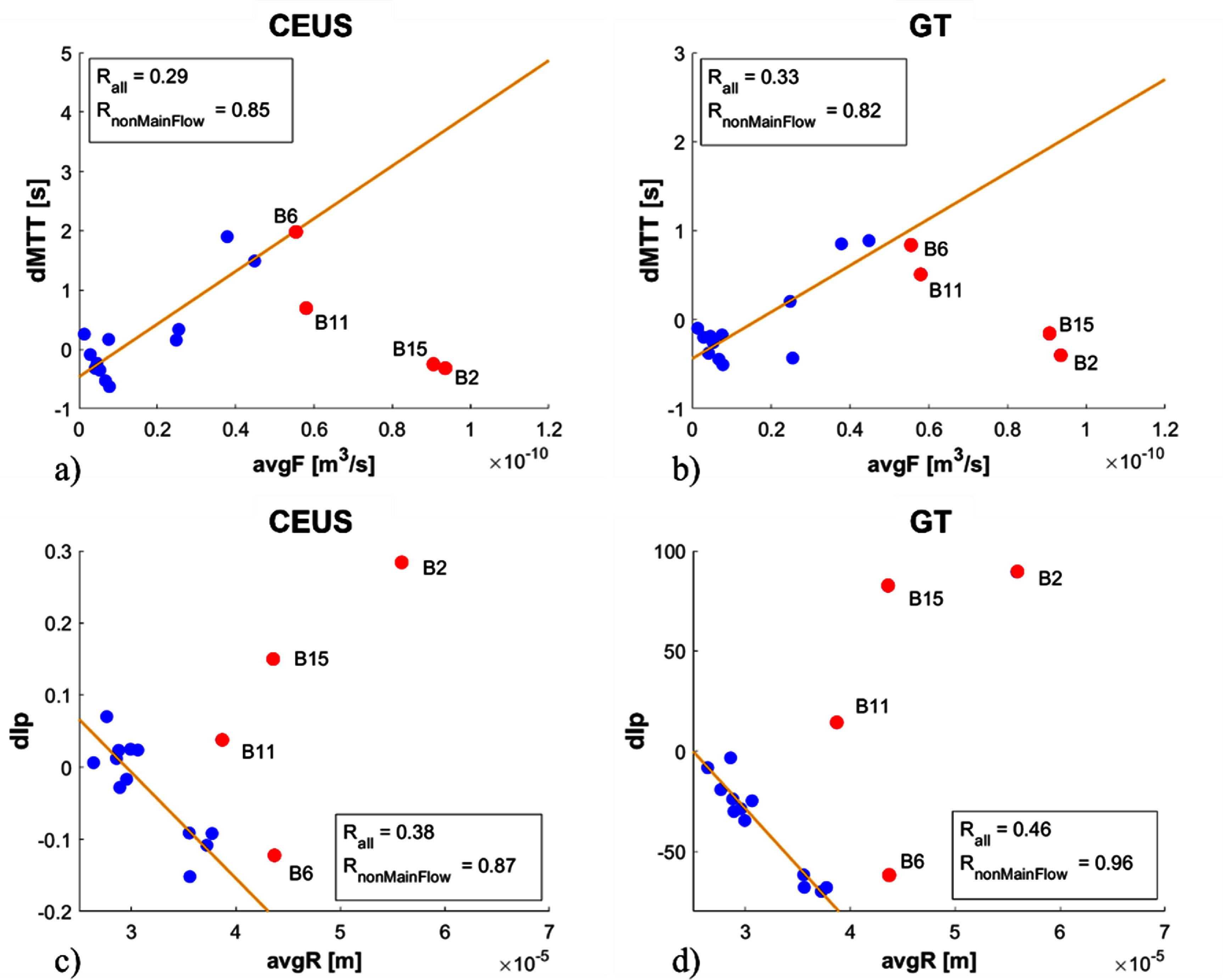
The difference of global parameter measurements compared to the base case is plotted against GT properties of the ROI that was removed, eg for the marker, **ROI11**, $d\textrm{MTT}$ = ($\textrm{MTT}_{wo\textrm{ROI}\textbf{B11}}-\textrm{MTT}_{\textrm{BN}}$) vs (mean Flow of **ROI11**). (a) $d\textrm{MTT}$ of CEUS against average flow of the removed ROI, (b) $d\textrm{MTT}$ of particle-time curve against average flow of removed ROI, (c) $dI\textrm{p}$ of CEUS against average radius of the removed ROI, and (d) $dI\textrm{p}$ of particle-time curve against average radius of removed ROI. Data in red corresponds to select ROIs on the main-flow path, $R_\textrm{nonMainflow}$ is the correlation upon omitting these main-flow cases. Data in blue corresponds to the remaining ROIs as presented in figure 2.

Here the change in $\textrm{TIC}$ parameters were calculated in order to assess the effect of occlusive ischaemia in the entire network. Given the relative nature of the $\textrm{TIC}$ parameters, the changes of these parameters in the entire network are calculated as a means of assessing the impact of an ischaemic ROI in the remainder network. The calculation for the $\textrm{MTT}$ change is defined as:

\begin{equation*} d\mathrm{MTT} = \mathrm{MTT}_{wo\mathrm{ROI}x} - \mathrm{MTT}_{\mathrm{BN}}\end{equation*}

where $\mathrm{MTT}_\mathrm{BN}$ is the $\mathrm{MTT}$ of the base, healthy network and $\mathrm{MTT}_{wo\mathrm{ROI}x}$ is the $\mathrm{MTT}$ of the remainder network once $\mathrm{ROI}x$ is ischaemic. The same formulation applies to other TIC parameters, including $dI\mathrm{p}$ that is applied here. Figure 6 plots $d\mathrm{MTT}$ against the flow of $\textrm{ROI}x$ for $\textrm{TIC}$ (figure 6(a)) and the $\textrm{TPC}$ (figure 6(b)). Both figures display correlation between $d\textrm{MTT}$ and original ROI flow in the cases of peripheral occlusion (displayed in blue, *R* = 0.85 and 0.82 for CEUS *TIC* and GT *TPC* respectively), while main-flow occlusion networks lack correlation. Main-flow ischaemias (**ROI2, ROI6, ROI11, ROI15** as seen in figure 2) do not overall affect $\textrm{MTT}$ in the total network, but they do generate higher average flow in the remaining network, resulting in loss of correlation between average flow and $d\textrm{MTT}$. This can be explained by the fact that a region of significant flow, thereby possessing a low $\textrm{MTT}$ value, is characterised by large vessel radii. Inducing occlusion in such a region effectively removes a significant channel through which MBs are able to flow and alternative paths do not entirely compensate in terms of transport. As a result, the global $\textrm{MTT}$ may or may not increase. Thus, the inlet and outlet regions which have the highest flow rates (**ROI2**, **ROI15**) present outliers to the general trend observed in figures 6(a) and (b). It is important to note that the CEUS and GT data agree and thus, similarly to the previous section it can be concluded that a significant flow redistribution is not possible to assess using $\textrm{MTT}$ changes of a large area and macroscopically.

Figures 6(c) and (d) plot $dI\textrm{p}$ against mean radius of the vessels of the removed region. $dI\textrm{p}$ possesses the best correlation with GT data as compared to other intensity parameters like $\textrm{AUC}$. Figures 6(c) and (d) plot the $\textrm{TIC}$ and $\textrm{TPC}$ $dI\textrm{p}$ respectively. The excellent negative correlation in both scatter plots refers to the removal of peripheral regions (*R* = 0.87 for CEUS $\textrm{TIC}$ and *R* = 0.96 for GT $\textrm{TPC}$) and stems from the fact that the average intensity of the remaining network, once an ROI is removed, will increase depending on the volume of the remainder network as the flow is redistributed. In other words, the larger the volume of the removed ROI, the smaller the volume of the remainder network, and as a result $dI\textrm{p}$ will correlate negatively to the volume of the removed ROI.

On the other hand, regions removed from the main-flow path also have large average vessel radii, but in this case the flow redistribution is significant and the remainder network increases capacity particularly for the highly significant inlet (**ROI2**) and outlet (**ROI15**). This suggests that like transit, intensity related parameters are also not good indicators of volume in real networks. The ischaemia/flow redistribution experiments here suggest that such parameters are not adequate in providing relative measures of volume or flow in the case of major ischaemia.

### 3.4. Super-resolution analysis

**Figure 7.**
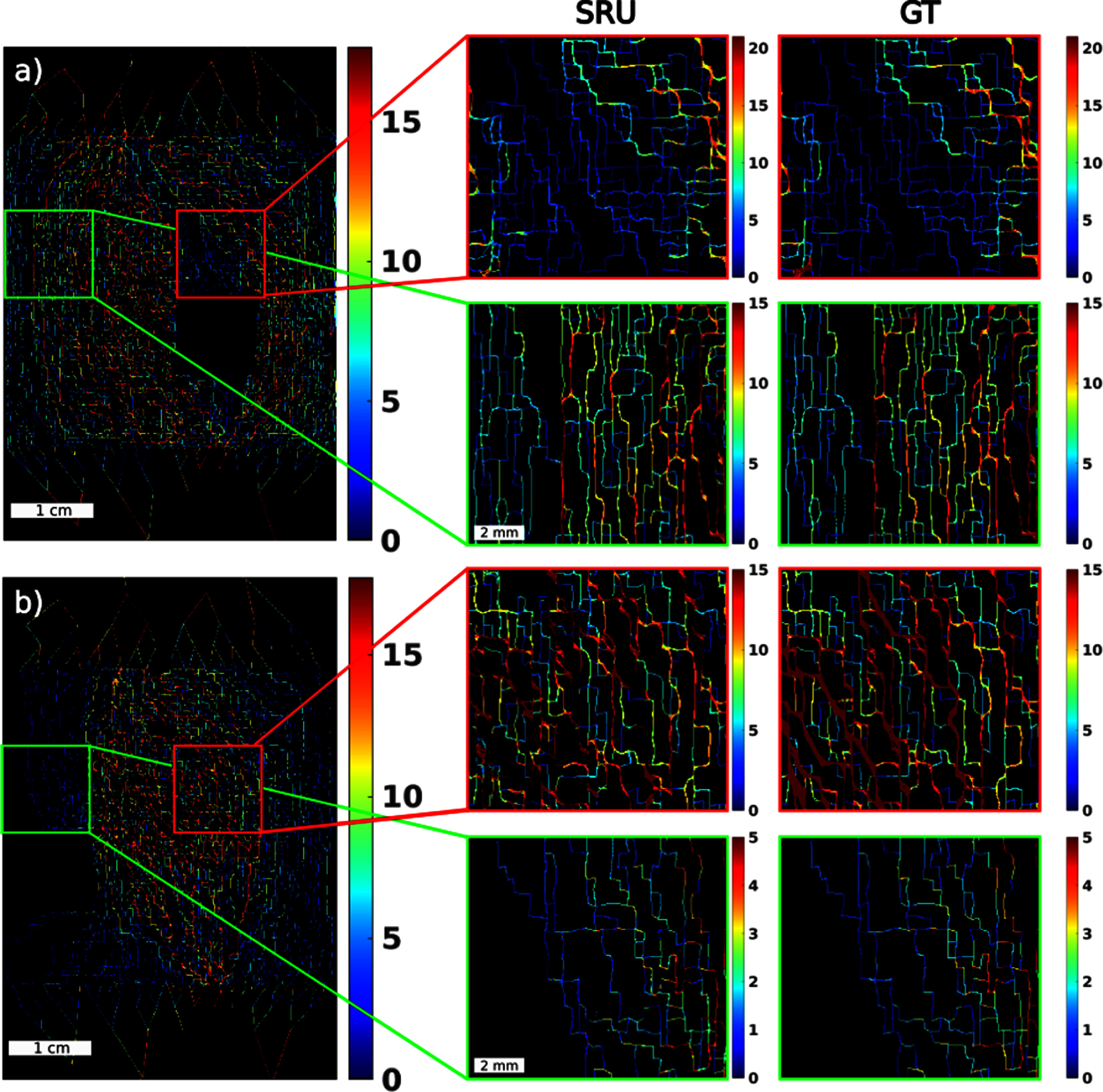
SRU velocity maps with the corresponding GT map for the main flow (ROI6 removed) occlusion case. In red is a main flow region and in green is a peripheral-flow region. The correspondence of structural recovery with the GT map via detections is 93.8% for the main flow region, and 91.3% for the peripheral flow region. (b) Equivalent map for the peripheral flow (ROI8 removed) occlusion case. Here, the correspondence of structure with the GT map is 93.3% for the main-flow region, and 93.9% for the peripheral flow region.

Localisation and tracking algorithms can provide maps in super-resolution such as for MB density, track density and velocity. These were produced in two key locations. Figure 7(a) displays velocity maps for main-flow occlusion (ROI6 removed) and figure 7(b) shows peripheral-flow occlusion (ROI8 removed). Here, for each occlusion case, the analysis focusses on inspecting two ROI types, in red a main-flow region (ROI10 inspected) and in green a peripheral-flow (ROI12 inspected). These regions are chosen as they are directly downstream of the removed regions and in each removal case they represent regions where the flow is either reduced or increased via flow redistribution.

The microvascular features of main-flow are apparent in the maps (figure 7), as well as details due to flow remodelling in the presence of an obstruction in a region of main-flow occlusion or reduction of flow in the vicinity of a peripheral-flow occlusion. SRUs capacity to recover vessel structure was measured via overlaying a detection skeleton (pixels that register detections) for each region against the GT, the correspondence was found to be $ > 91\%$ in all the zoomed regions of figure 7, which demonstrates that each SRU map depicts structural network detail accurately.

**Figure 8.**
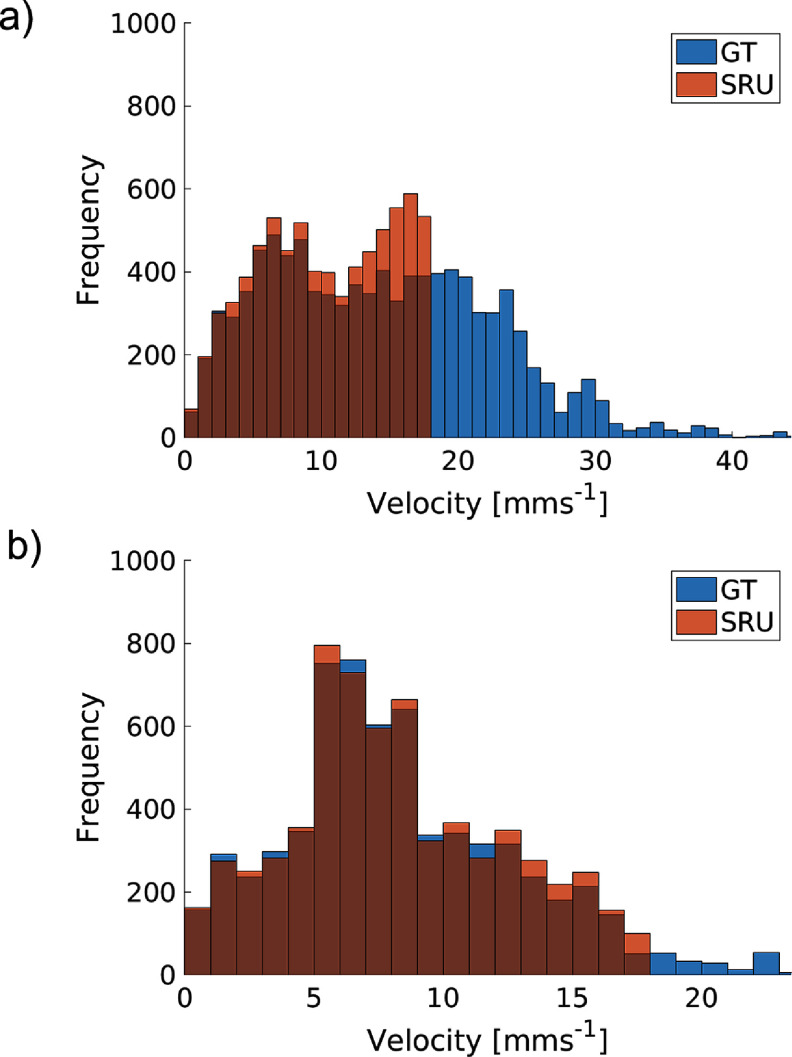
Histogram GT-SRU comparison of the two flow redistribution inspections. (a) Peripheral- flow occlusion, main-flow inspection. (b) Main-flow occlusion, peripheral-flow inspection.

Histogram analysis enables a quantitative assessment (figure 8). Figure 8(a) represents main-flow region velocity distribution for the peripheral occlusion.

Here there is an accurate replication of velocities up to 18 mm s^−1^, while velocities above this value are not registered in the SRU maps due to a thresholding imposed as an input parameter. Figure 8(b) represents peripheral-flow region velocity distribution for the main-flow occlusion, showing an identical pattern of capturing accurately velocities up to 18 mm s^−1^, and failing to record velocities above this value, with the caveat that this flow case results in overall much lower flow rates, so the impact on correlation to the GT is less significant. Table 3 shows the impact on the measurement of the mean vessel velocity of each region and the associated systematic uncertainty. Consistent with the above is the variation of the systematic uncertainty, as at different regions there is a different proportion of faster MBs. This is most significant when assessing the main-flow region, as even in the case of occluding proximal to the assessment region, the nature of being on the main-flow path results in the maximum velocity achieved in these cases being the highest.

**Table 3.** Mean GT velocity with relevant velocity correspondence between SRU and GT for each flow scenario.

		Mean GT velocity (mm s^−1^)	Mean SRU velocity (mm s^−1^)	over/under-estimation (+/-%)
Main-flow assessment	Main-flow occlusion	6.01	6.86	−12.39
	Peripheral-flow occlusion	10.22	14.48	−29.42

Peripheral-flow assessment	Main-flow occlusion	8.19	8.34	−1.8
	Peripheral-flow occlusion	2.1	1.99	+5.24

## 4. Discussion

CEUS has been the subject of decades of research from basic science and engineering to pre-clinical and clinical research. One of the main goals of the field was the quantitative assessment of tissue microvascularisation and perfusion, given that this will lead to diagnostic application related to a number of non-communicative diseases. However, there is currently no clinical implementation of tissue perfusion quantification. The limitations are well understood and attributed to operator dependence, contrast administration and patient related variabilities (Tang *et al* 2011, Averkiou *et al* 2020). Further, pre-clinical work has been shown that $\textrm{TIC}$ parameters, such as $\textrm{AUC}$, have inherent systematic uncertainties due to the object of imaging, which is often a dense network of vessels with ranging diameters (Butler *et al* 2019). Within the conventional resolution limit the image intensity is primarily determined by the largest vessels (even mm sized) and intensity that results from microvasculature is very difficult to deconvolve and thus quantify. This is attributed to a number of factors including microbubble nonlinear response, and also different transit kinetics in vessels even within a single pixel. The *in silico* study here provides new information on a CEUS limitation also inherent to macroscopic $\textrm{TIC}$ derivation, which is captured in the GT studies as well as in the CEUS simulation. Although an ischaemia is depicted in the images, it is the consequence of it in the remainder network flow redistribution that shows low correlations of $\textrm{TIC}$ parameters with actual vascular dynamics. This is primarily due to the varying degree of flow redistribution that depends on the location of the ischaemia, as well as the fact that a number of $\textrm{TIC}$ quantification assumptions do not hold, such as mass conservation for small regions and the single input/output (Strouthos *et al* 2010). This is relevant to cardiovascular disease as well as other diseases that provide local changes of flow such as cancer. In cardiovascular disease for example, the measurement of microvascular dynamics related indices, such as the development of collateral flow, that may provide diagnostic or prognostic biomarkers and impact patient management. The fundamental limitation of macroscopic CEUS perfusion measurements lies in individual pixel intensities that stem from an average scatter of MBs. These vessels may be highly variable, including arterial and venous flow in both macro- and microvessels. It is thus inevitable that transit related measurements, that incorporate both global and local dynamics, are limited in revealing microvascular function that is relevant to a specific pathophysiology. SRU is an imaging modality that originates from CEUS and promises to revolutionise medical imaging. Typically, it relies on tracking individual contrast MBs as they travel in the blood stream, which results in delineation (or ‘painting’) of the host vessels. It can thus provide direct measurements of microvascular anatomy and function down to the arteriolar level (20–50 $\mu\mathrm{m}$ diameter vessels (Christensen-Jeffries *et al* 2020)), representing a leap in resolution compared to any state-of-the-art imaging techniques (MRI, CT, ultrasound, and PET), that only provide resolution in the millimetre range. More importantly, and as evidenced here, maps produced by SRU imaging contain direct information at microvascular level (architecture, vessel dynamics), that may be quantified and may well prove relevant to the detection and diagnosis of a large number of noncommunicable diseases. The entire flow redistribution that results from ischaemia is accurately depicted and at appropriate resolution. Similar level microvascular changes, e.g. collateral flow, vasodilatation, vasoconstriction, apart from mapping functioning vessel dynamics, are expected to be possible to measure. Opposite to the conventional CEUS TIC quantification, that appears inherently limited to depict vascular remodelling and direct perfusion quantification, SRU can depict individual microvessels directly, offering a capability beyond the measurement of perfusion as expressed by macroscopic imaging. SRU will likely generate disease-specific multi-parametric biomarker development that draws from relevant structural, architectural and dynamic features, a departure from cumulative biomarker types as is the case for CEUS and perfusion. However, further work will be required to develop SRU biomarkers that have diagnostic or prognostic capability for ischaemic tissue as, to our knowledge, there are no such studies in the literature. SRU velocity results display several under- and over-estimations when compared to GT values figure 3. The algorithm limitation to depict the higher velocity vessels is expected as the algorithm here deploys nearest neighbour tracking in its simplified form (Kanoulas *et al* 2019). As a result, MBs are tracked up to a maximum velocity set as a chosen displacement threshold, which manifests in under-estimation of average velocities against ground-truth particle speeds in high-flow vessels (figure 8(a)). Increasing this threshold may improve tracking of high speed MBs, but at the expense of erroneous track generation. The thresholds here were chosen to achieve correct tracking for the majority of MBs. Note that, erroneous tracks generate artefactual vessels. Missed tracks lead to missing vessels or misrepresentation of flow. A number of attempts to address the correct depiction of microvascular dynamics are increasingly found in the literature (Ackermann and Schmitz 2016, Song *et al* 2018, Tang *et al* 2020). The optimal current solution is presented by grouping MBs depending on their dynamic behaviour (velocity, direction) (Huang 2020). Another limitation is associated with the over-estimation of velocity registered in the peripheral-flow region where the occlusion is proximal, representing the lowest flow scenario studied. This relates to the overestimation of minimum MB velocity, mainly attributed to the localisation error and the pixel size of the original data. However, super-resolution techniques provide image analysis and sub-pixel resolution capabilities to overcome this. The value chosen here was deemed appropriate for the dimensions and dynamics of the model. Finally, the data acquisition protocol has a finite duration and microbubble number. This may lead to less than 100% coverage of the vascular network. In other words, it is unlikely that all the vessels in view will be crossed by a microbubble, and thus be displayed in the SRU maps. While this may be recognised as a reduction of information, it is its impact on a specific diagnostic biomarker under investigation that is of importance. Thus, data acquisition protocols should be linked directly to and guided by the clinical value. Note, that the current SRU processing requires clinical acquisition times in the order of minutes with additional immobilisation to ensure same plane scanning, which remains challenging. It is unclear to what degree SRU is impacted by motion, and certainly current methods are not optimised to disentangle intricacies of complex tissue motion. This may in the future be alleviated or resolved if there is adequate 3D information for motion correction and/or significant reduction of acquisition time. However, while motion correction is implemented by several SRU methodologies in the literature it is important to note that there are several diagnostic applications including cancer, such as the prostate (Kanoulas *et al* 2019), and cardiovascular disease, such as peripheral artery disease (Meneses *et al* 2018), that provide minimal motion and where a secured ultrasound probe can help collect high quality data. Detailed knowledge of SRU deployment without aspects such as tissue motion, as conducted here, is an essential precursor and motivator to eventually discern the impact of these factors and the engineering required to overcome them. The study here demonstrates that SRU is in principle capable of depicting directly microvascular changes. It is important to note that the comparison between SRU and CEUS is not made using direct physical quantities. For example, the area overlap is relevant for SRU as direct assessment of vessel delineation capacity, while the CEUS area is a rather saturated area of vascular signal (Lassau *et al* 2006) with little impact and it has been demonstrated that $\textrm{TIC}$ derived parameters are more sensitive to perfusion changes (Lassau *et al* 2010, Chen *et al* 2023). Likewise, deriving transit metrics from SRU derived vessel velocity measurements is a redundant exercise as already the GT data shows their limitations discussed above. Therefore, the comparison between CEUS and SRU is done at fundamental level in terms of their capacity to depict microvascular function and kinetics. Finally, one of the study’s main limitations is the unrealistic nature of the network, including its architecture, distribution of vessels sizes and dynamics. Real 3D networks have typically different structures and dynamics. For example, venous and arterial flow are spatially adjacent, which is not possible to provide in 2D. Further, the uncertainties introduced by the 2D processing of a 3D volume (Butler *et al* 2019) are absent. However, these limitations are of little importance here, as the comparison is performed on fundamental capabilities of CEUS and SRU analysis. Indeed, the main aim is to use the simulation of the vascular effect of flow redistribution and enable a good comparison between an ideal $\textrm{TIC}$ with the respective GT, which was achieved. Note, that an *in vivo* derived bolus flow pattern as expressed by a realistic haemodynamic model is preserved. Further, it is expected that CEUS measurements are likely to be further compromised by a full 3D investigation, due to the network changes mentioned above, while the SRU quantification fundamentally differs little to the 2D as it refers to the measurement of each identified vessel. The development of 3D microvascular models will be required in future, however, to fully address the development of diagnostic or prognostic biomarkers that are relevant to specific pathophysiology, understanding the uncertainty will be required.

The use of an artificial network is justified for the purposes of this study through the concomitant structure and flow that underpin the flow of particles, scattering MBs in this case. Controlled construction and application of proper boundary conditions allow for network dependent pressure gradients from inlet to outlet, in which the presence of the active vessel region dictates the assigned flows to each vessel and thus the velocities. Control of the studied model provides the platform for a supervised simulation of occlusion, whereby the responses to restricted vasculature is accurately imparted to the particles as they traverse the system. Applications of ultrasound related modalities to scanned models have been investigated. However, it is highly challenging to address their full vascularisation in silico. This is often because first a full 3D model would be most realistic and such models do not have the vascular bed in its entirety in 3D or lack the rigorous construction required to generate particle flow that can be tied to changes in the network. To overcome this, models of this kind have simulated flow by distributing MBs within the network and assigning velocities based on *in vivo* data (Belgharbi *et al* 2023) and not as a result of an established flow gradient pertaining to specific network structure and pressure environment. Aside from a divergence in flow assignment, disconnecting particle behaviour from tractable pressure and flow gradients prevents induction of network changes to simulate vascular affliction or disease. Thus, modality interaction with perturbations to flow scenarios against a healthy case is prevented. Further, the simulation of an entire 3D vascular system that includes vessel scales from arteriolar scale to the microvascular bed, representing a realistic model, is not available in the literature. This is significant, as it is reported that in the case of severe ischaemia, collateral arterioles are responsible for reperfusion and thus recovery (Maddedu *et al* 2006). Moreover, collateral flow recovery can often occur from considerably further upstream than the occlusion site. Current studies of flow compatible models are conducted on systems of brain microvasculature with maximum vessel radius ($ < 55\,\mu\mathrm{m}$, with mean radii ∼10$\,\mu\mathrm{m}$) not representing major flow-contributing collaterals associated with recovery, nor extending further than 0.65 mm in any direction (Boujelben *et al* 2016, Belgharbi *et al* 2023). Development of the methodology presented here is therefore justified through encapsulation of parameters relevant to ischaemia in an initial, simplistic scenario, which isolates imaging quantification from the nuances introduced by model realism. Thereby initiating a mutual model-modality development platform that generates well-documented and rigorous knowledge of SRU’s performances for a given model. From this, the creation of gradually more complex models, moving towards more clinically relevant cases on realistic, 3D vessel networks, is facilitated with a back catalogue of previous performance expectations distinguishing the presence of realistic biomarkers from imaging uncertainty, guiding advancement of pathology modelling specific to SRU.

## Data Availability

The data cannot be made publicly available upon publication because they contain commercially sensitive information. The data that support the findings of this study are available upon reasonable request from the authors.
